# Resource-aware whole-cell model of division of labour in a microbial consortium for complex-substrate degradation

**DOI:** 10.1186/s12934-022-01842-0

**Published:** 2022-06-14

**Authors:** Eliza Atkinson, Zoltan Tuza, Giansimone Perrino, Guy-Bart Stan, Rodrigo Ledesma-Amaro

**Affiliations:** Department of Bioengineering and Imperial College Centre for Synthetic Biology, Imperial College London, London, SW72AZ UK

**Keywords:** Microbial consortia, Division of labour, Resource-aware whole-cell modelling, Consolidated bioprocesses, Synthetic biology

## Abstract

**Background:**

Low-cost sustainable feedstocks are essential for commercially viable biotechnologies. These feedstocks, often derived from plant or food waste, contain a multitude of different complex biomolecules which require multiple enzymes to hydrolyse and metabolise. Current standard biotechnology uses monocultures in which a single host expresses all the proteins required for the consolidated bioprocess. However, these hosts have limited capacity for expressing proteins before growth is impacted. This limitation may be overcome by utilising division of labour (DOL) in a consortium, where each member expresses a single protein of a longer degradation pathway.

**Results:**

Here, we model a two-strain consortium, with one strain expressing an endohydrolase and a second strain expressing an exohydrolase, for cooperative degradation of a complex substrate. Our results suggest that there is a balance between increasing expression to enhance degradation versus the burden that higher expression causes. Once a threshold of burden is reached, the consortium will consistently perform better than an equivalent single-cell monoculture.

**Conclusions:**

We demonstrate that resource-aware whole-cell models can be used to predict the benefits and limitations of using consortia systems to overcome burden. Our model predicts the region of expression where DOL would be beneficial for growth on starch, which will assist in making informed design choices for this, and other, complex-substrate degradation pathways.

**Supplementary Information:**

The online version contains supplementary material available at 10.1186/s12934-022-01842-0.

## Background

### Agricultural waste as low-cost feedstock for biotechnology

Existing methods for biotechnology primarily use processed sugar feedstocks, which are high-cost and non-sustainable. Recycling agricultural waste for use as cheap and sustainable feedstocks for microbes will enable more cost-effective bioproduction, capable of competing with traditional industrial practices. To achieve this requires organisms engineered to hydrolyse and metabolise the complex biomolecules found in plants, such as lignin, cellulose and starch. Many attempts have been made to engineer desirable production into naturally lignocellulolytic or saccharolytic hosts, or to engineer substrate hydrolysis into optimised industrial production hosts [1, 2]. However, both methods have failed to achieve the high yields needed for a viable commercial process in a single host monoculture [3, 4]. One of the primary limitations to a consolidated bioprocess in a single host has been attributed to the finite energy pool and high interference between the components and with the host [5–7].

### Burden and division of labour

The energetic and resource cost of expressing heterologous proteins is known as gene expression burden. In the model organism *Escherichia coli* this burden is primarily attributed to the energetic cost of translation elongation and inefficient use of translational resources [8, 9]; for example, ribosomes wastefully sequestered on transcripts during ribosomal traffic jams. The result is a limited expression level of heterologous genes that can be achieved in a single cell before the burden causes decreased growth rates and reduced product yields [10, 11].

**Fig. 1:**
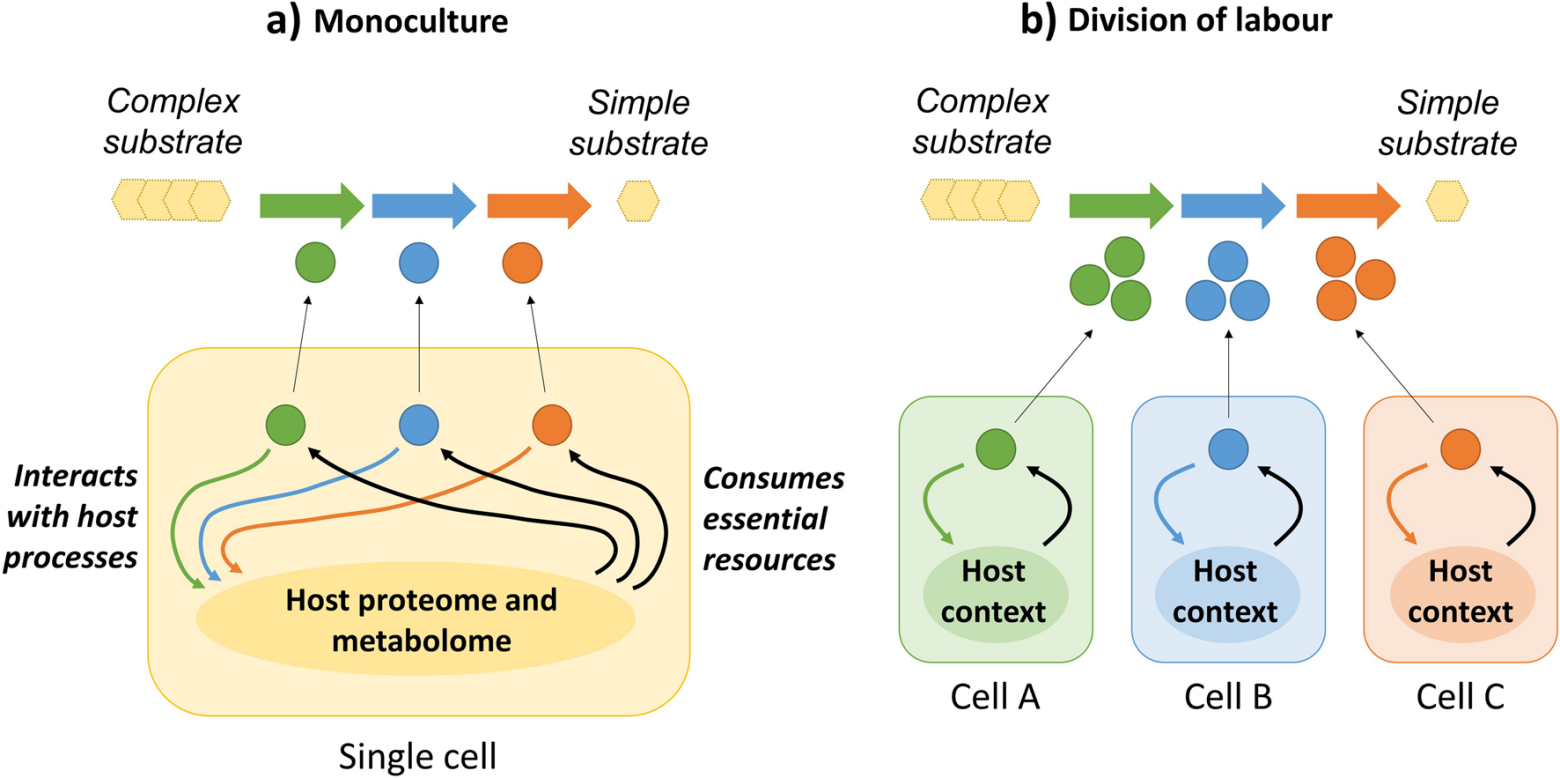
Division of labour for complex substrate degradation. **a** In monoculture all heterologous components of a degradation pathway are expressed in a single cell. This typically causes significant burden by consuming limited cellular resources and interfering with essential host bioprocesses. **b** In a multicellular system the labour is divided between cells, reducing the burden on each individual cell. Expression in each cell can be optimized in parallel and the ratio of the different cells can be used to control flux through each step of the pathway

One method to reduce the burden of heterologous gene expression is division of labour (DOL) between members of a microbial consortium (Fig. 1) [12, 13]. Since fewer proteins need to be expressed in each cell, competition between heterologous and endogenous genes for shared cellular resources is decreased [14, 15]. DOL has been used for the production of a wide range of useful molecules, including anthocyanins [16], flavonoids [17], *n*-butanol [18], 3-amino-benzoic acid [19] and tryptamine [20].

Importantly, DOL may assist in the design of consolidated bioprocesses. Efforts to engineer utilization of lignocellulosic biomass into desirable bioproduction hosts have been complicated by the number and complexity of the degradation enzymes required. DOL can overcome the limitations of these design efforts by reducing the burden imposed on each cell and enabling an alternative means of optimising the expression of each gene in parallel. For example, Tsai, Goyal & Chen engineered four different strains of *Saccharomyces cerevisiae*, each expressing a different subunit of the cellulosome. Altering the ratio of the four strains in the consortium allowed them to optimise the processes of cellulose hydrolysis and ethanol production [4, 21, 22].

### DOL and computational modelling

Computational models can assist in the design of DOL strategies. A broad range of approaches are used for this, including genome-scale metabolic models (GEMs) [23] and agent-based models [24, 25]. Of interest here, are coarse-grained modelling approaches called host-aware models, which consider the relationship between heterologous protein expression and host cell growth. These models have been used to identify key factors enabling co-culture to outperform monoculture [26, 27]. For example, modelling has assisted in the design of a glucose-acetate cross-feeding consortium by identifying the range of burden within which the consortium is stable [27, 28]. Tsoi et al*.* [29] used a phenomenological approach to model 24 different pathway architectures for DOL, which allowed them to identify when burden is significantly limiting in the monoculture and when the cost of transporting intermediates becomes a limiting factor for consortia.

Most coarse-grained models of consortia use simple phenomenological relationships between increasing expression and decreasing growth, treating the mechanisms behind this relationship as a black/grey box. In contrast, more mechanistic host-aware models for monocultures have been proposed which explicitly model the intracellular trade-offs and resource allocations that characterise burden. This includes the dynamics for shared cellular resource pools such as ribosomes [30], global cellular feedback to regulate protein production [31], and external regulation from varying environments [32]. Coarse-grained models which account for cellular resource-pathway interactions within the cell can predict non-intuitive effects of heterologous expression on the host. These resource-aware models may offer advantages over the current phenomenological models used for consortium growth predictions but have yet to be applied to contexts involving more than one host strain.

Weiße et al. published a host-aware whole-cell model for *E. coli* which links transcription and translation to the allocation of cellular energy, ribosomes, and the proteome [33]. This model has been widely used to predict burden for genetic circuits [34, 35]. Here, we adapt this model to predict the growth rates achievable by a simple consortium designed to perform complex-substrate degradation. Many complex polysaccharide substrates found in plants, such as starch, cellulose, xylan or inulin, require two enzymes for efficient degradation: an endohydrolase to cleave bonds within the molecule into smaller chunks and an exohydrolase to release sugars from the end of polysaccharide chains. This simple pathway can be expressed with two genes in a single cell or in a two-strain consortium where each strain expresses one of the genes. Here we use the example of starch hydrolysis to investigate a consortium of two strains of *E. coli,* expressing separately endoamylase and exoamylase. Cultured together, these strains should be able to grow on starch as the sole-carbon source. This model allows exploration of the impact of burden on this two-strain consortium and comparison with a monoculture co-expressing both hydrolytic enzymes simultaneously.

## Results

### A resource-aware whole-cell model for burden

**Fig. 2:**
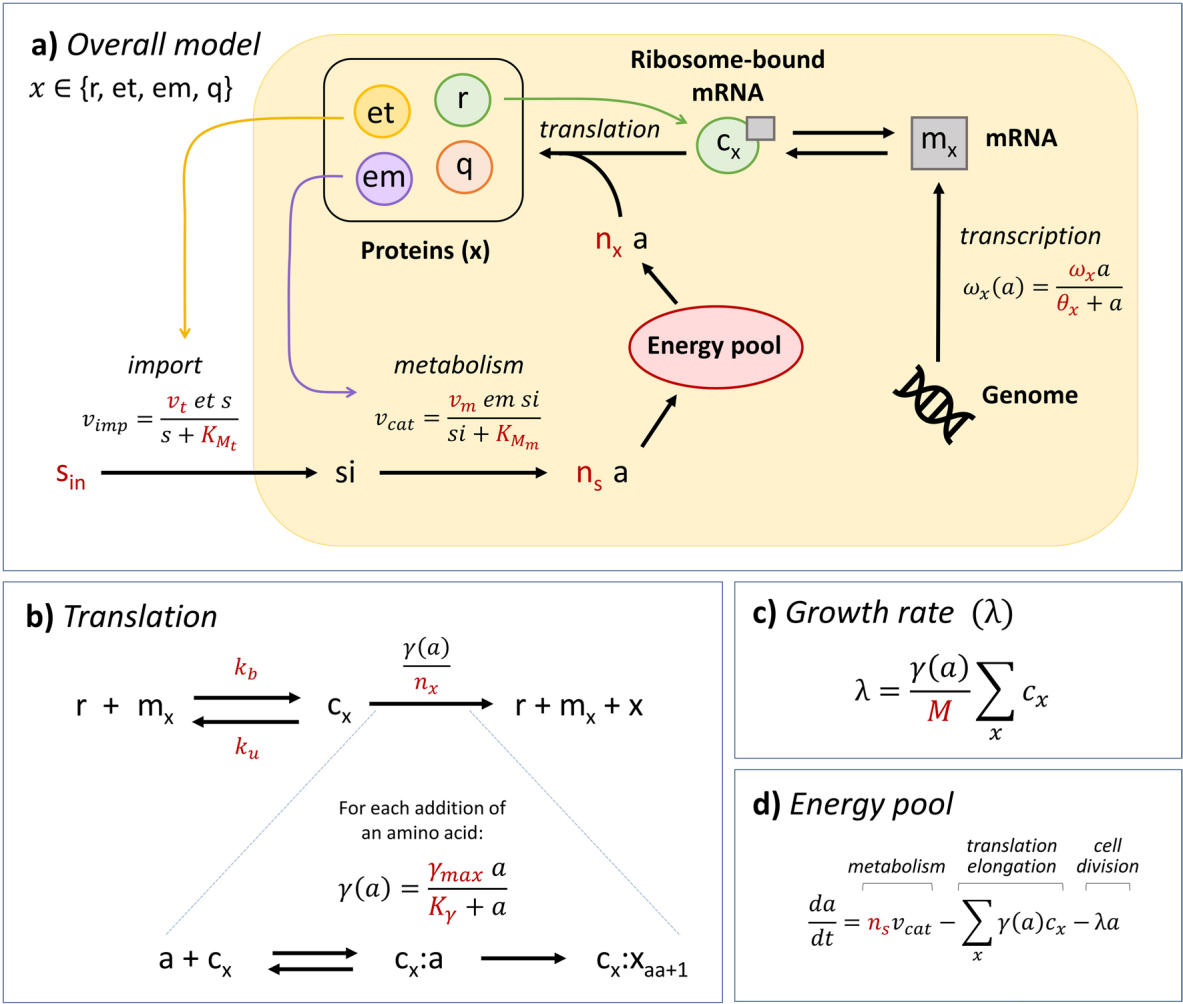
Weiße’s whole-cell modelling framework. **a** Expression of proteins is carried out in 3 steps; transcription, ribosome binding and translation. Free mRNA, ribosome-bound mRNA and protein are denoted by **m**_**x**_, **c**_**x**_ and **x** respectively, with **x** being** r** for ribosomal, **q** for housekeeping, **em** for metabolic or **et** for transport. The substrate, **s**, is supplied to the cell at a constant rate (s_in_) controlled by a chemostat and imported into the cell by transport proteins. The imported substrate, **si**, is converted into energy units, **a**, by metabolic proteins. **b** A simplified translation model accounts for ribosome binding and unbinding to mRNA and then an irreversible elongation reaction. Energy units are consumed in translation elongation, with one energy unit consumed for each addition of an amino acid (aa). **c** Growth rate, **λ**, is calculated as a function of the rate of translation of all proteins in the cell and the total proteome content of the cell. All intracellular molecules will be lost at a dilution rate equal to the growth rate. **d** The finite energy pool available to the cell is determined by the balance between energy produced by metabolism of the substrate and consumption of energy required for protein translation. For a full description of these equations, see Weiße et al. [33] and Additional file 1: Table S1. Parameters (in red): **x ∈ {r:** ribosomal, **et:** transport, **em:** metabolic, **q:** housekeeping}; **s**_**in**_**:** rate of glucose input; **v**_**t**_: k_cat_ of transport reaction; **K**_**Mt**_: Michaelis constant for transport reaction; **v**_**m**_: k_cat_ of metabolic reaction; **K**_**Mm**_: Michaelis constant for metabolic reaction; **n**_**s**_: nutrient efficiency; **ω**_**x**_: maximum transcription rate of protein **x**; **θ**_**x**_: “transcription threshold” for protein **x; γ**_**max**_: maximum translation rate; **K**_**γ**_: “translation threshold”; **k**_**b**_: rate of ribosome binding; **k**_**u**_: rate of ribosome unbinding; **n**_**x**_: length of **protein x** in amino acids; **M**: all amino acids in the proteome

Firstly, a whole-cell model for burden was adapted to predict cellular resource allocation and growth rate when expressing heterologous proteins in a DOL system. The ordinary differential equations (ODEs) describing this whole-cell model are derived from those published by Weiße et al. [33] (Fig. 2, Additional file 1: Table S1). The full details of the assumptions made and how the model equations are obtained can be found in Weiße et al. [33], but the key features of the model are:

1. The proteome is roughly divided into 4 categories, ribosomal proteins (r), transport proteins (et), metabolic proteins (em) and housekeeping proteins (q) as described in Fig. 2a. Each of these 4 categories of proteins have ODEs for 3 species of intracellular molecules; the mRNA (m_x_), the mRNA:ribosomal complex (c_x_) and the protein (x), where x ∈ {r, et, em, q}. It is assumed that all types of mRNA degrade at the same rate, and that the proteins are not degraded.
2. A further 2 ODEs model the imported substrate (si) and the energy molecules (a), equivalent to ATP. The primary substrate (s) is provided at a constant rate. Extracellular substrate is imported into the cell by transport proteins. The imported substrate is then converted into energy molecules by metabolic proteins. The number of energy units produced from each substrate is dependent on the nutrient efficiency (n_s_) (Fig. 2d).
3. The model assumes that translation accounts for the total energy use in the cell. To model translation, each free mRNA binds to a ribosome from the finite pool of free ribosomes, and each addition of an amino acid requires the use of one unit of energy (Fig. 2b).
4. At each time point, growth rate is calculated based on a set of linear growth laws described by Scott et al*.* [9] (Fig. 2c).

This model was adapted to a two-strain consortium; where one cell (Cell A) expresses enzyme A (ea) and the other (Cell B) expresses enzyme B (eb). For each cell type (A or B) an ODE model was created capturing the dynamics of the 14 intracellular molecules considered in Weiße’s framework, plus 3 new ODEs capturing the dynamics of the mRNA, the mRNA:ribosome complex and the protein for each heterologous gene. The equations for ribosomes and growth rate were adjusted to account for the new heterologous proteins as outlined in Nikolados et al. [36]. To enable comparison with the monoculture case, a model was similarly built by adding 6 ODES to the model of Weiße et al. to capture co-expression of both enzymes A (ea) and B (eb) in a single cell. The same parameters employed by Weiße et al. for an *E. coli* host cell were used [33].

An important consideration when adapting the model to the consortium was the necessity to provide substrate for both Cell A and Cell B. The rate of uptake of glucose from the environment into the cells, ${v}_{uptake}\left(et,s\right)$, was adjusted to account for the number of each cell type in the consortia, (see Eq. 1), or in the monoculture (see Eq. 2). For the purpose of this model, we assume that the populations of both cell types in the consortium can be tightly controlled. This could be achieved by a number of different methods; by computationally-controlled addition of each cell type in a turbidostat, or by further engineering of the cells with autoinhibition or cross-feeding such that the populations will autoregulate themselves to remain at a stable ratio [37–40].

1 $${v}_{uptake}\left(et,\,s\right)= \frac{{v}_{et}\, s ({N}_{a}\, e{{t}_{cell}}_{a}+{N}_{b}\, e{{t}_{cell}}_{b})}{s + {K}_{{m}_{et}}}$$

2 $${v}_{uptake} \left(et,s\right)= \frac{{v}_{et}\, s\, N et}{s + {K}_{{m}_{et}}}$$

Here ${N}_{a}$ and ${N}_{b}$ are the number of Cell A and Cell B respectively, and $N$ is the number of cells in the monoculture population. For the case study presented here we assume that the two cell types in the consortium remain stable in equal ratio and that the populations can be normalised so the ratio of Cell A to Cell B is 1:1. For a fair comparison between the consortium and monoculture cases, we assume there is an equal total number of cells in both cases. Therefore, if the value of ${N}_{a}$ and ${N}_{b}$ are each 1, an equivalent monoculture will have $N$ equal to 2. In this framework, $et$ is the number of transport proteins per cell, which may vary across Cell A and Cell B, while ${v}_{et}$ is the k_cat_ of the transport reaction catalysed by all the transport proteins in the cell, and ${K}_{{m}_{et}}$ is the Michaelis constant for the same transport reaction.

The amount of glucose available to the cells, $s$, will depend on the glucose input, ${s}_{in},$ the amount of glucose consumed by the all the cells in the population, ${v}_{uptake}(et,s)$, and the dilution rate, ${d}_{s}$, of the chemostat, modelled using Eq. 3:

3 $$\frac{ds}{dt}={s}_{in}-{v}_{uptake}(et,s)-{ d}_{s} s$$

All parameter values used in the model are summarised in Additional file 1: Table S2.

### DOL reduces resource redistribution to heterologous genes

**Fig. 3:**
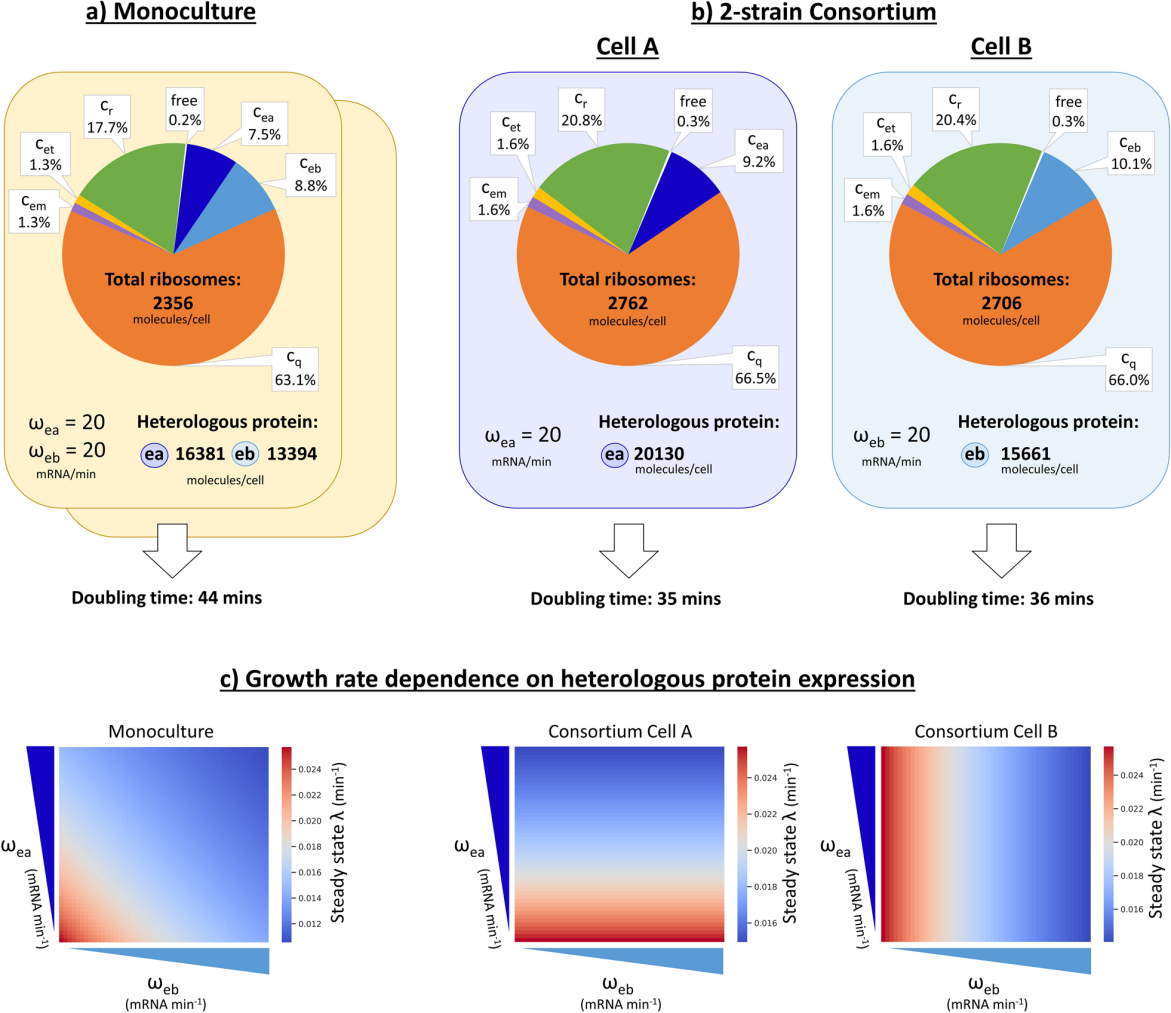
Simulation results for division of labour (DOL) compared to single-cell co-expression. Our proposed model accounts for the number of free ribosomes (**free**), and the number of ribosomes engaged in translation (**c**_**x**_) for the production of different types of protein (**x**, where **x** is **ea**, heterologous protein A; **eb**, heterologous protein B; **q**, housekeeping protein; **em**, metabolic enzyme; **et**, transport enzyme; **r**, ribosomal protein). Two heterologous proteins, **ea** and **eb**, can be expressed in a monoculture or consortium. Considering maximum transcription rates (**ω**) of 20 mRNA/min for both proteins, we use this model to predict the steady-state amount of ribosomes dedicated to the translation of these proteins and the associated cell growth rate that results from the reallocation of these ribosomes. **a** In monoculture at steady state 16.3% of ribosomes are diverted to heterologous expression with an associated doubling time of 44 min. **b** DOL in a two-strain consortium means the resources of each cell are only diverted to the expression of a single protein. The overall reallocation of ribosomes to heterologous expression is thus reduced. However, the fraction of ribosomes allocated to the translation of each protein type is higher in the consortium case (ea: 9.2%, eb: 10.1% in the consortium case, compared to 7.5% and 8.8%, respectively in the monoculture case). The doubling time is faster compared to the monoculture case, while the number of heterologous proteins of each type produced per cell is higher. **c** In monoculture, the growth rate (**λ**) is dependent on the maximum transcription rates (**ω**) of both heterologous proteins. In the consortium, the growth rate is dependent on only one of the two transcription rates. **ea** & **eb**: two generic heterologous proteins**, c**_**x**_: ribosome:mRNA complexes for protein x (**x** ∈ {**ea**, **eb**, **q**, **em**, **et**, **r**}), **λ**: growth rate, **ω**_**ea**_: maximum transcription rate of ea, **ω**_**eb**_: maximum transcription rate of eb

The whole-cell model we briefly introduced above can be used to investigate burden when two heterologous proteins are expressed using DOL and compare the simulation results with those obtained from co-expressing all heterologous proteins in a single cell. Running a simulation with glucose as the primary substrate shows that DOL in a two-strain consortium composed of Cells A and B achieves faster growth rates than the monoculture case (Fig. 3a and b). As the maximum transcription rate (ω) of the expressed heterologous proteins increases, the growth rate decreases. In the monoculture, growth rate is thus dependent on the transcription rate of both enzyme A and enzyme B (Fig. 3c), highlighting competition for resources between heterologous proteins and host processes. In the consortia, however, the growth rate of each cell (Cell A or Cell B) depends on the transcription rate of the single heterologous protein it expresses.

The model predicts that DOL will improve the amount of heterologous protein that can be produced per cell. Cell A produces 20,130 molecules/cell of enzyme A (7.95% of total protein in Cell A; Additional file 1: Table S3), while Cell B produces 15,661 molecules/cell of enzyme B (6.34% of total protein in Cell B). In contrast a single cell in the monoculture produces 16,381 enzyme A and 13,394 enzyme B (11.94% of total protein). While the total number of proteins in the Weisse model may be underestimated [41, 42], the values for heterologous gene expression as a percentage of total protein lie within an acceptable range as *E. coli* has been shown to tolerate up to 30% of their proteome dedicated to heterologous expression [43].

While in the consortia, each cell type, A or B, can produce more of its respective heterologous protein per cell than the monoculture, this may not result in higher heterologous protein yield overall. In a monoculture of 1000 cells, all 1000 will express both proteins. In a consortium with 1000 cells, 500 will express protein A, and 500 will express protein B. Assuming that each cell in these populations produces the amount of protein predicted by the whole-cell model; a 1000-cell monoculture will produce around 16 million enzyme A and 13 million enzyme B. While a 1000-cell consortium will only produce around 10 million enzyme A and 8 million enzyme B. Therefore, while the monoculture case has slower growth and produces fewer heterologous proteins per cell, it may produce more heterologous proteins in total than the equivalent consortia case.

### Starch-degradation

Starch was identified as a desirable complex substrate for the application of division of labour. Starch is the second most abundant source of carbon in plants after cellulose and a common by-product of the food industry. Starch granules are made of two polymers of glucose, amylose and amylopectin, which differ in their structure and glycosidic bonds. Efficient degradation of starch requires both endo- and exo- amylase activities to break down the two types of glycosidic bonds found in its structure. α-amylase (EC 3.2.1.1) is an endo-amylase which binds randomly in the sugar chain and catalyses hydrolysis of α-1,4 glycosidic bonds between glucose units, creating variable-length maltodextrins [44]. Glucoamylase (EC 3.2.1.3) is an exo-amylase which releases glucose from the non-reducing ends of a sugar chain by cleaving either α-1,4 or α-1,6 bonds [44]. These two enzymes are relatively well characterized for several different starch-sources and maltodextrins.

Mathematically modelling the degradation of starch by amylase can be complex when the parallel activity of both amylases and the stochasticity of bond cleavage are taken into account [45, 46]. Therefore this model utilized a simplified set of equations proposed by Fujii and Kawamura [47]. When starch is abundant, its degradation is modelled using a two-step process. The α-amylase-dependent first reaction produces an intermediate which can act as a substrate for the glucoamylase-dependent second reaction. The reasoning for this schema is that at the beginning of starch hydrolysis there are relatively few non-reducing ends within the whole molecule that glucoamylase can act on, so the dominant reaction is the α-amylase hydrolysis reaction. Similarly, once a chain is degraded to a certain length, or limited by branches, α-amylase affinity for the substrate decreases. If a large proportion of starch is degraded to short chains, then the α-amylase reaction becomes negligible and the glucoamylase rate parameters will change based on the average chain length of the polysaccharide, as described in Fujii and Kawamura [47]. Both α-amylase and glucoamylase show Michaelis–Menten kinetics in terms of the rate of hydrolysis. Equations 4 and 5 below show the rate of change for starch, $s0$, and the intermediate, $s1$, respectively. When starch is externally supplied as the primary substrate, glucose, $s$, will vary based on Eq. 6, replacing the previously stated Eq. 3 which is only applicable when glucose is supplied as the primary substrate.

4 $$\frac{ds0}{dt}={s{0}_{in} - N}_{a}\frac{{v}_{ea}\, ea\, s0 }{s0+{K}_{{m}_{ea}}} -{d}_{s0}\mathrm{ s}0$$

5 $$\frac{ds1}{dt}={N}_{a}\frac{{v}_{ea}\, ea\, s0}{s0+{K}_{{m}_{ea}}} - {N}_{b}\frac{{v}_{eb}\, eb\, s1 }{s1+{K}_{{m}_{eb}}}-{d}_{s1}\mathrm{ s}1$$

6 $$\frac{ds}{dt}={N}_{b}\frac{{v}_{eb}\, eb\, s1 }{s1+{K}_{{m}_{eb}}}-{v}_{uptake}(et,s)-{ d}_{s} s$$

**Fig. 4:**
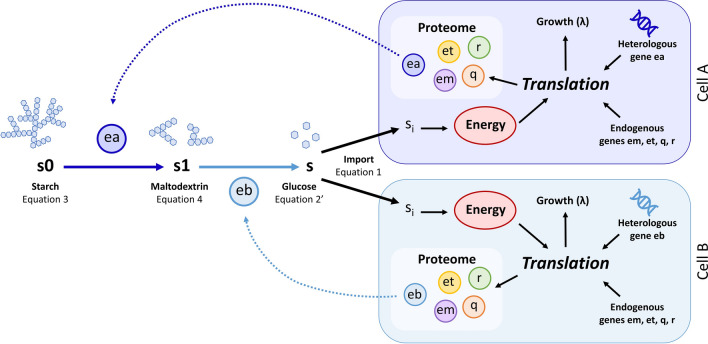
Model for starch utilisation with division of labour in a two-strain consortium. Each cell is modelled using the Weiβe modelling framework adapted to account for the additional expression of either α-amylase (**ea**) or glucoamylase (**eb**). These amylases each catalyse a step in the degradation of starch. The first step is an α-amylase-dependent reaction, which degrades starch (**s0**) and produces maltodextrin intermediates of various lengths (**s1**). These intermediates are then degraded to glucose (**s**) via a glucoamylase-dependent reaction. Glucose is imported into each cell and the intracellular molecules of the two cells are modelled with the whole-cell model based on Weiβe et al*.* [33] (Fig. 2)

Here $ea$ represents the concentration of α-amylase, while $eb$ denotes that of glucoamylase, ${v}_{ea}$ and ${v}_{eb}$ represent the k_cat_ rates for $ea$ and $eb$, respectively, and ${K}_{{m}_{ea}}$ and ${K}_{{m}_{eb}}$ are the Michaelis constant for each enzyme. The rate of each step is also dependent on the number of cells expressing α-amylase (i.e. the population of Cell A (${N}_{a}$)) and the number of cells expressing glucoamylase (i.e. the population of cell B (${N}_{b}$)). For a monoculture system all cells will express both, therefore ${N}_{a}$ and ${N}_{b}$ in Eqs. 4, 5 and 6 will be replaced with $N$ (Additional file 1: Table S4). In this example, we assume that each population type can be tightly controlled and that the proportion of Cell A to Cell B can be robustly maintained at a 1:1 ratio. Under this assumption, without loss of generality, we consider ${N}_{a}$ = 1, ${N}_{b}$ = 1 and $N$ = 2, so that the monoculture and consortium have equal total number of cells. The rate of starch input, $s{0}_{in}$, and the rate of loss of each substrate, ${d}_{s0}$, ${d}_{s1}$ and ${d}_{s}$, can be adjusted for the desired bioreactor setup. These equations were combined with the ODEs for Cell A and Cell B as shown in Fig. 4.

### DOL can achieve higher growth rate on starch than an equivalent monoculture system

To investigate the relationship between amylase expression, starch degradation and burden when starch is used as the primary carbon source, we use the model in Eqs. 1–5 to numerically simulate growth rate for a range of expression levels of amylases.

**Fig. 5:**
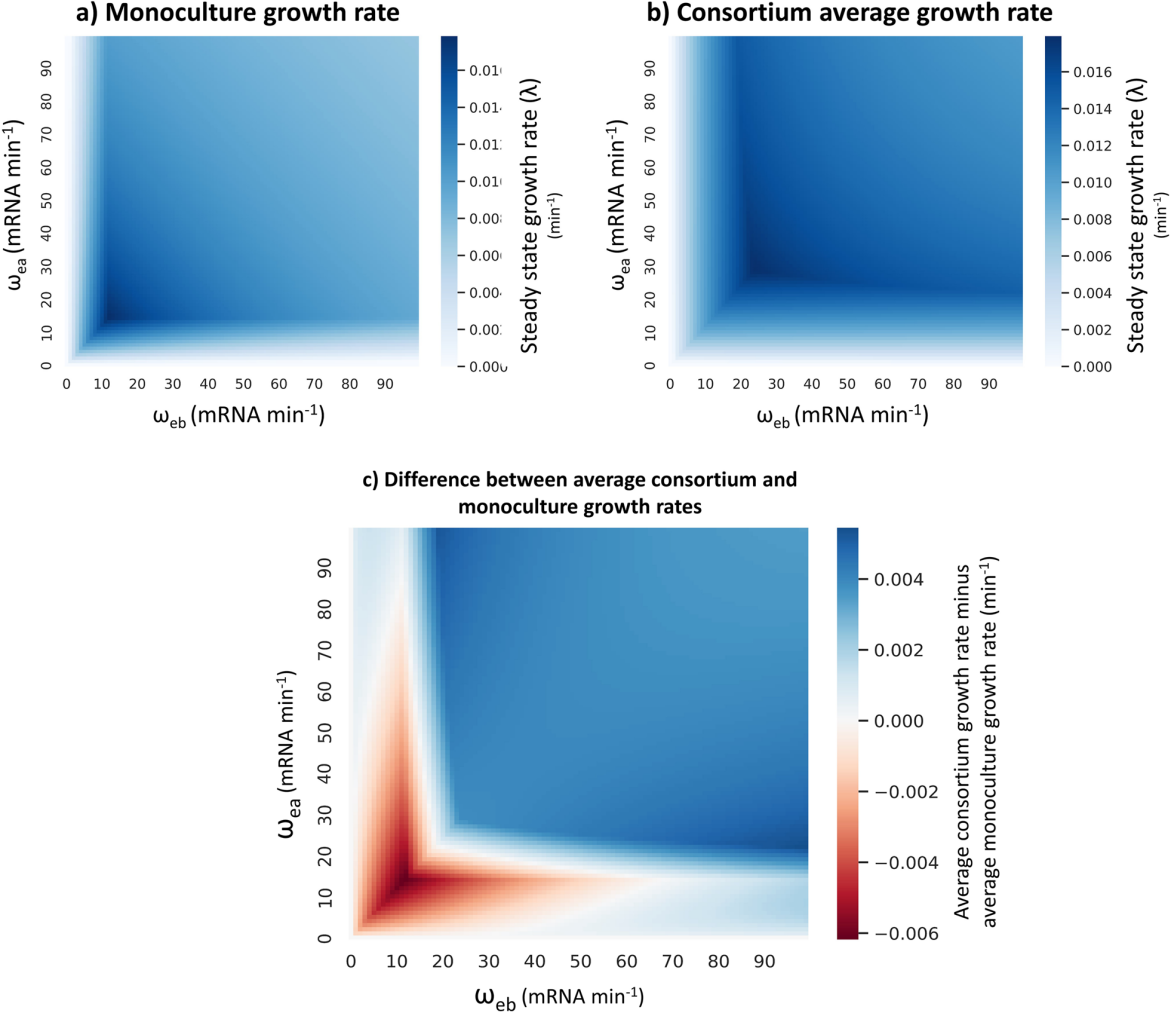
Changes in the steady state growth rate with varying transcription rates for α-amylase (ea) and glucoamylase (**eb**), in monoculture and division of labour (DOL) systems of expression. Changes in growth rate (λ, min^−1^) for an average cell in a monoculture or a two-strain consortium, as the maximum transcription rates of α-amylase and glucoamylase (**ω**_**ea**_ and **ω**_**eb**_ respectively) are varied. To obtain biologically realistic values for each parameter combination, a numerical simulation with glucose as the main carbon source was first run until steady-state values were reached. Using these steady-state values as initial conditions, we then performed a second run with starch as the main carbon source. **a** Steady-state growth rate of an average cell co-expressing α-amylase and glucoamylase. **b** Steady-state growth rate of an average cell in the consortium. Cell A expresses α-amylase, while Cell B expresses glucoamylase. The consortium growth rate is calculated as the average growth rate of the two cells. **c** Comparison of monoculture and consortium growth rate (difference measured as the average growth rate of the consortium minus the monoculture growth rate)

Figure 5a shows the growth rate for a monoculture single cell as a function of the maximum transcription rate (ω) of α-amylase (ea) and glucoamylase (eb). Similarly, Fig. 5b shows the average growth rate of the cells in the consortium, where one cell (Cell A) expresses only α-amylase, and the other (Cell B) expresses only glucoamylase. The maximum growth rate achieved by the monoculture was 0.01784 min^−1^, or a doubling time of 38.85 min, which was achieved at ω_ea_ = 15 and ω_eb_ = 12 mRNA min^−1^. Different ratios of the two cell types in the consortium were tested. Approximately equal ratios showed the best average growth rate for the consortium (Additional file 1: Fig. S1). For a 1:1 ratio of the cell types the maximum average growth rate achieved by the consortium was 0.01793 min^−1^, or a doubling time of 38.66 min, which was achieved at ω_ea_ = 29 and ω_eb_ = 24 mRNA min^−1^. At this maximum, Cell A is growing at 0.01779 min^−1^ (doubling time 38.96 min), while Cell B is growing at 0.01807 min^−1^ (doubling time 38.36 min).

Considering the cell types in the consortium separately, the fastest growth rate achieved for Cell A in the consortium was 0.01915 min^−1^ (doubling time 36.20 min), at ω_ea_ = 22 mRNA/min and ω_eb_ = 99 mRNA/min (Cell B at these values has a growth rate of 0.01004 min^−1^ or a doubling time of 69.04 min). The fastest growth rate for Cell B was 0.01924 min^−1^ (doubling time 36.03 min), at ω_ea_ = 99 mRNA/min and ω_eb_ = 19 mRNA/min (Cell A at these values has a growth rate of 0.01098 min^−1^ or a doubling time of 63.13 min). This shows that the growth of each cell type in the consortium is maximal when its consortium partner’s transcription rate is high.

Figure 5c shows the difference between the average consortium growth rate and the monoculture growth rate. At lower transcription rates the monoculture has a faster growth rate than the consortium (red region in Fig. 5c). We assume a minimal population of 2 cells in both the monoculture and the consortium. In the monoculture, we assume each of the 2 cells produces both the heterologous proteins in the amount predicted by the monoculture whole-cell model. In the consortium, however, assuming a 1:1 ratio of the two cell types, each cell only produces one of the heterologous proteins. Based on the whole-cell model predictions a 2-cell monoculture will produce a higher number of amylases in total than a 2-cell consortium (Additional file 1: Fig. S2). Interrogating the different regions in Fig. 5c reveals that the parameter values of the red region, at which the monoculture grows better than the consortium, correspond to the parameter values at which the monoculture is consuming all the glucose provided by the amylases (Additional file 1: Fig. S3). This indicates that when glucose production is the limiting factor for growth (rather than heterologous gene expression burden) the monoculture system has the advantage over the DOL system.

At higher transcription rates, the consortium outperforms the monoculture (blue region in Fig. 5c). At these parameter values the monoculture is more affected by burden than the consortium since it co-expresses two heterologous proteins which divert resources away from essential endogenous genes. At the higher transcription levels, this allows the consortium to achieve a higher maximum growth rate than the monoculture. Burden must be sufficiently limiting to growth, which will occur at the higher transcription levels, for DOL to be an appropriate strategy to improve growth on starch. Changing the ratio of the two cell types will shift the threshold at which the consortium outperforms the monoculture (Additional file 1: Fig. S4).

## Discussion

Whole-cell models for predicting burden have been underutilised in the design of DOL in consortia. Here, we have demonstrated that these models can be adapted to observe and predict the difference in growth when expressing heterologous genes by DOL versus in a single cell. A consortium of two cells each expressing a single heterologous protein achieves a higher growth rate than a monoculture co-expressing two heterologous proteins; this benefit of DOL is a phenomenon often observed in natural microbiomes. By explicitly modelling the resource allocations of translation this model reflects the biological mechanism behind this observation; the redirection of ribosomes toward heterologous gene expression is greater in the monoculture cell than in either of the consortium cells. It is important to note that, in the monoculture, the growth rate and production of both enzymes is dependent on both genes’ expression level, which reflects a design challenge when trying to optimise either of the two genes in a one-factor-at-a-time approach. In the DOL case, when grown on glucose, each cell is only dependent on the transcription rate of its respective protein, meaning both cells could be optimised independently and in parallel. Because of the reduced burden, either cell of the consortium, Cell A or Cell B, produces more per cell of their respective enzyme A or B than the amount produced per cell in the monoculture. However, at a population level, when the monoculture and the consortium both have the same total number of cells, the monoculture will produce a higher total amount of heterologous proteins than the consortium. Therefore, it is important to consider the end-point application of such DOL. If the aim is to produce the maximum of enzyme A or enzyme B then the monoculture may be preferable, whereas if the aim is to provide optimal growth rates, perhaps for production of an endogenous molecule of interest, then DOL may be preferable.

Here, the application of interest is in dividing the labour of complex-substrate degradation, with starch used as a specific example. Whether or not DOL is suitable to improve growth of engineered *E. coli* on starch depends on the balance between useful expression of the amylases to produce glucose, and non-productive expression which creates unnecessary burden. The model identifies the parameter space within which the monoculture or the DOL system is preferable. Where starch degradation to glucose is the limiting factor to growth, a monoculture system would be preferable. A 2-cell monoculture performs better than a 2-cell consortium at low transcription rates because total amylase production is higher when 2 cells are co-expressing both amylases, compared to a duoculture consortium in which each cell is expressing a single type of amylase. However, there is a threshold beyond which amylase expression is no longer beneficial to growth and instead becomes detrimental. Monoculture cells reach this threshold at a lower transcription rate than the consortium cells. For all transcription rates above this threshold the consortium achieves a higher growth rate than the monoculture. Therefore, where burden is the limiting factor to growth, a DOL system is preferable. The consortium could also achieve a higher maximum growth rate than the monoculture if the parameters are optimised precisely. By identifying the parameter space within which DOL can provide benefits, this model will inform design choices for complex-substrate degradation and aid in attaining the optimum expression level for maximal growth.

When grown on glucose as the primary carbon source, the difference in growth achieved by the consortia compared to the monoculture is much more distinct, because the expressed heterologous protein is disconnected from the substrate production. Currently the only output is growth on starch, however for a full consolidated bioprocess, the cells should also biosynthesise desirable products. Heterologous biosynthesis genes will not have the positive connection to growth seen by amylases. Therefore, DOL may be more beneficial to a bioproduction pathway than to a substrate degradation pathway.

One important limitation of this model is that it assumes a stable ratio of cell types in the consortium and therefore does not consider the effect of asymmetric growth rates on population dynamics. In the monoculture the primary substrate is shared equally, as all cells grow at the same rate. But, in consortia, if one cell type has a faster growth rate it may consume more substrate than its consortium partner. The current version of the two-strain consortium model indicates that if one protein transcription is set very high and the other very low there may be a significant difference in growth rate between the two cell types. The lower-expressing cell will have a survival advantage and could dominate a co-culture. However, this model assumes that populations can be robustly controlled at a stable fixed ratio and therefore does not give any perspective on the population dynamics. Simplified host-aware coarse-grained models or agent-based model approaches may be preferable for achieving both cellular-level and population-level dynamics.

Another limitation of the model in its current state is the lack of experimental validation for the quantitative results. This model demonstrates the reduced metabolic burden that is seen when DOL is implemented. This advantage of microbial consortia is supported by experimental data for DOL in biosynthesis pathways, allowing higher productivity or production of molecules that is impossible to achieve by single cells [16, 17, 48]. Further, there are many experimental studies that support the benefits of using consortia for degradation of lignocellulosic biomass [21, 49–51], including combining α-amylase-expressing species with glucoamylase-expressing species [52]. However, experimental studies which directly compare a consortium expressing different degradation enzymes with an equivalent co-expressing monoculture are currently an underexplored area of investigation. So, while this study provides a theoretical prediction for how such a consortium would behave compared to monoculture, these predictions remain to be verified experimentally.

Currently, our proposed approach only considers gene expression burden caused by the energetic cost and reallocation of translational resources. However, burden can encompass other costs incurred by a host cell due to the expected or unexpected functions of the synthetic pathway [53]. When introduced to a new proteome and metabolome, heterologous proteins may be promiscuous [54]. This can cause toxicity burden, and includes the production of unexpected toxic byproducts, induction of unfolded-protein stress response, or obstruction of endogenous protein function [11]. On the other hand, the heterologous protein may function as desired but inadvertently produce role-based burden, such as depleting the intracellular resources for native metabolic pathways [53]. Therefore, some pathways that could benefit from DOL due to these other burdens will not accurately be predicted by our proposed model.

## Conclusions

In conclusion, we have proposed a resource-aware whole-cell model that highlights the interesting relationships that exist between DOL and burden for a system where primary substrate catabolism is dependent on heterologous protein production. A two-strain *E. coli* consortium expressing α-amylase and glucoamylase singularly in different cell types can achieve a higher growth rate on starch than a monoculture co-expressing both genes. However, this is only achievable within a particular region of expression, identified by the model, where amylase production is not limiting the glucose supplies to the cell. This model may be reparametrized to other host organisms where translation plays the primary role in heterologous expression burden. Further, adapting this, or similar, resource-aware coarse-grained models may be useful in the future for other DOL applications, such as distributed biosynthesis pathways or multicellular genetic circuits, in predicting burden and informing design choices.

## Methods

### Hardware and software

Data was generated on a Windows 10 PC with Intel® Core™ i7-1065G7 CPU @ 1.30 GHz, 16 GB RAM using Python 3.7 with a Spyder 4.1 (as part of Anaconda) IDE. Where multiple processors were available, the computational labour was divided between processors using the dask.distributed package (distributed.dask.org) for Python.

### ODE solver and simulation methods

The scipy.integrate.solve_ivp function from the scipy package was used to solve ODEs, with the LSODA method (Adams/BDF method with automatic stiffness detection and switching). In all simulations the time span was set to 0–20,000 min, and the absolute tolerance (atol) and relative tolerance (rtol) were set to 1e^−12^. Steady state was taken as the value for each intracellular molecule at the end of a numerical simulation of 20,000 min.

For simulations where glucose is supplied externally the ODEs were initialised with 1000 energy units (a) and 10 ribosomes (r), as in the original Weiße model. For simulations on starch, a simulation was first run for growth on glucose, then the steady-state values reached were used as initial conditions for a simulation on starch.

## Supplementary Information

**Additional file 1.** Additional Tables and figures.

## Data Availability

The model generated during this study is available publicly on the GitHub repository: https://github.com/elizaatkinson/Resource-aware-whole-cell-model-of-DOL-in-2-strain-consortium-for-complex-substrate-degradation.

## References

[1] Olguin-Maciel E, Singh A, Chable-Villacis R, Tapia-Tussell R, Ruiz HA (2020). Consolidated bioprocessing, an innovative strategy towards sustainability for biofuels production from crop residues: an overview. Agronomy.

[2] Fan Z. Consolidated bioprocessing for ethanol production. Biorefineries Integr Biochem Process Liq Biofuels. 2014. p. 141–60.

[3] Brethauer S, Studer MH (2014). Consolidated bioprocessing of lignocellulose by a microbial consortium. Energy Environ Sci.

[4] Minty JJ, Lin XN. Engineering synthetic microbial consortia for consolidated Bioprocessing of ligonocellulosic biomass into valuable fuels and chemicals. Direct Microb Convers Biomass to Adv Biofuels. Elsevier; 2015. p. 365–81.

[5] Brenner K, You L, Arnold FH (2008). Engineering microbial consortia: a new frontier in synthetic biology. Trends Biotechnol.

[6] Verbeke TJ, Zhang X, Henrissat B, Spicer V, Rydzak T, Krokhin OV (2013). Genomic evaluation of *Thermoanaerobacter* spp. for the construction of designer co-cultures to improve lignocellulosic biofuel production. PLoS ONE.

[7] Tsai SL, Oh J, Singh S, Chen R, Chen W (2009). Functional assembly of minicellulosomes on the *Saccharomyces cerevisiae* cell surface for cellulose hydrolysis and ethanol production. Appl Environ Microbiol.

[8] Scott M, Hwa T (2011). Bacterial growth laws and their applications. Curr Opin Biotechnol.

[9] Scott M, Gunderson CW, Mateescu EM, Zhang Z, Hwa T (2010). Interdependence of cell growth and gene expression: origins and consequences. Science.

[10] Borkowski O, Ceroni F, Stan G-B, Ellis T (2016). Overloaded and stressed: whole-cell considerations for bacterial synthetic biology. Curr Opin Microbiol.

[11] Glick BR (1995). Metabolic load and heterologous gene expression. Biotechnol Adv.

[12] Chen T, Zhou Y, Lu Y, Zhang H (2019). Advances in heterologous biosynthesis of plant and fungal natural products by modular co-culture engineering. Biotechnol Lett.

[13] Zhang H, Wang X (2016). Modular co-culture engineering, a new approach for metabolic engineering. Metab Eng.

[14] Bernstein HC, Paulson SD, Carlson RP (2012). Synthetic Escherichia coli consortia engineered for syntrophy demonstrate enhanced biomass productivity. J Biotechnol.

[15] Zhang H, Pereira B, Li Z, Stephanopoulos G, Demain AL (2015). Engineering *Escherichia coli* coculture systems for the production of biochemical products. Proc Natl Acad Sci USA.

[16] Jones JA, Vernacchio VR, Collins SM, Shirke AN, Xiu Y, Englaender JA (2017). Complete biosynthesis of anthocyanins using *E. coli* polycultures. MBio.

[17] Jones JA, Vernacchio VR, Sinkoe AL, Collins SM, Ibrahim MHA, Lachance DM (2016). Experimental and computational optimization of an *Escherichia coli* co-culture for the efficient production of flavonoids. Metab Eng.

[18] Saini M, Hong Chen M, Chiang CJ, Chao YP (2015). Potential production platform of *n*-butanol in *Escherichia coli*. Metab Eng.

[19] Zhang H, Stephanopoulos G (2016). Co-culture engineering for microbial biosynthesis of 3-amino-benzoic acid in *Escherichia coli*. Biotechnol J.

[20] Wang X, Policarpio L, Prajapati D, Li Z, Zhang H (2020). Developing *E. coli*-*E. coli* co-cultures to overcome barriers of heterologous tryptamine biosynthesis. Metab Eng Commun..

[21] Tsai SL, Goyal G, Chen W (2010). Surface display of a functional minicellulosome by intracellular complementation using a synthetic yeast consortium and its application to cellulose hydrolysis and ethanol production. Appl Environ Microbiol.

[22] Goyal G, Tsai SL, Madan B, DaSilva NA, Chen W (2011). Simultaneous cell growth and ethanol production from cellulose by an engineered yeast consortium displaying a functional mini-cellulosome. Microb Cell Fact.

[23] Thommes M, Wang T, Zhao Q, Paschalidis IC, Segrè D (2019). Designing metabolic division of labor in microbial communities. mSystems..

[24] Gutiérrez M, Gregorio-Godoy P, Pérez Del Pulgar G, Munoz LE, Sáez S, Rodríguez-Patón A (2017). A new improved and extended version of the multicell bacterial simulator gro. ACS Synth Biol.

[25] Matyjaszkiewicz A, Fiore G, Annunziata F, Grierson CS, Savery NJ, Marucci L (2017). BSim 2.0: an advanced agent-based cell simulator. ACS Synth Biol.

[26] Rollié S, Mangold M, Sundmacher K (2012). Designing biological systems: systems engineering meets synthetic biology. Chem Eng Sci.

[27] Harvey E, Heys J, Gedeon T (2014). Quantifying the effects of the division of labor in metabolic pathways. J Theor Biol.

[28] Mauri M, Gouzé JL, de Jong H, Cinquemani E (2020). Enhanced production of heterologous proteins by a synthetic microbial community: conditions and trade-offs. PLoS Comput Biol.

[29] Tsoi R, Wu F, Zhang C, Bewick S, Karig D, You L (2018). Metabolic division of labor in microbial systems. Proc Natl Acad Sci USA.

[30] Gorochowski TE, Avcilar-Kucukgoze I, Bovenberg RAL, Roubos JA, Ignatova Z (2016). A minimal model of ribosome allocation dynamics captures trade-offs in expression between endogenous and synthetic genes. ACS Synth Biol.

[31] Liao C, Blanchard AE, Lu T (2017). An integrative circuit-host modelling framework for predicting synthetic gene network behaviours /631/553/552 /631/553/2695 article. Nat Microbiol.

[32] Sickle JJ, Ni C, Shen D, Wang Z, Jin M, Lu T (2020). Integrative circuit-host modeling of a genetic switch in varying environments. Sci Rep.

[33] Weiße AY, Oyarzún DA, Danos V, Swain PS (2015). Mechanistic links between cellular trade-offs, gene expression, and growth. Proc Natl Acad Sci USA.

[34] Darlington APS, Kim J, Jiménez JI, Bates DG (2018). Dynamic allocation of orthogonal ribosomes facilitates uncoupling of co-expressed genes. Nat Commun.

[35] Nikolados EM, Weiße AY, Ceroni F, Oyarzún DA (2019). Growth defects and loss-of-function in synthetic gene circuits. ACS Synth Biol.

[36] Nikolados E-M, Weiße AY, Oyarzún DA, Oyarzún O. Prediction of cellular burden with host-circuit models. 2020. http://arxiv.org/abs/2004.00995 10.1007/978-1-0716-1032-9_13 33405227

[37] Annunziata F, Matyjaszkiewicz A, Fiore G, Grierson CS, Marucci L, Di Bernardo M (2017). An orthogonal multi-input integration system to control gene expression in *Escherichia coli*. ACS Synth Biol.

[38] Losoi PS, Santala VP, Santala SM (2019). Enhanced population control in a synthetic bacterial consortium by interconnected carbon cross-feeding. ACS Synth Biol.

[39] McCardell RD, Huang S, Green LN, Murray RM (2017). Control of bacterial population density with population feedback and molecular sequestration. bioRxiv..

[40] Jiang W, Yang X, Gu F, Li X, Wang S, Luo Y (2022). Construction of synthetic microbial ecosystems and the regulation of population proportion. ACS Synth Biol.

[41] Soufi B, Krug K, Harst A, Macek B (2015). Characterization of the *E. coli* proteome and its modifications during growth and ethanol stress. Front Microbiol.

[42] Milo R (2013). What is the total number of protein molecules per cell volume? A call to rethink some published values. BioEssays.

[43] Dong H, Nilsson L, Kurland CG (1995). Gratuitous overexpression of genes in *Escherichia coli* leads to growth inhibition and ribosome destruction. J Bacteriol.

[44] El-Fallal A, Abou M, El-Sayed A, Omar N (2012). Starch and microbial α-amylases: from concepts to biotechnological applications. Carbohydrates.

[45] Bijttebier A, Goesaert H, Delcour JA (2008). Amylase action pattern on starch polymers. Biologia.

[46] Azhari R, Lotan N (1991). Enzymic hydrolysis of biopolymers via single-scission attack pathways: a unified kinetic model. J Mater Sci Mater Med.

[47] Fujii M, Kawamura Y (1985). Synergistic action of α-amylase and glucoamylase on hydrolysis of starch. Biotechnol Bioeng.

[48] Zhang H, Li Z, Pereira B, Stephanopoulos G (2015). Engineering *E. coli*-*E. coli* cocultures for production of muconic acid from glycerol. Microb Cell Fact.

[49] Kogo T, Yoshida Y, Koganei K, Matsumoto H, Watanabe T, Ogihara J (2017). Production of rice straw hydrolysis enzymes by the fungi *Trichoderma reesei* and *Humicola insolens* using rice straw as a carbon source. Bioresour Technol.

[50] Li X, He Y, Zhang L, Xu Z, Ben H, Gaffrey MJ (2019). Discovery of potential pathways for biological conversion of poplar wood into lipids by co-fermentation of Rhodococci strains. Biotechnol Biofuels.

[51] Chen L, Du JL, Zhan YJ, Li JA, Zuo RR, Tian S (2018). Consolidated bioprocessing for cellulosic ethanol conversion by cellulase–xylanase cell-surfaced yeast consortium. Prep Biochem Biotechnol.

[52] Wang S, Tang H, Peng F, Yu X, Su H, Xu P (2019). Metabolite-based mutualism enhances hydrogen production in a two-species microbial consortium. Commun Biol.

[53] Ellis T (2019). Predicting how evolution will beat us. Microb Biotechnol.

[54] Cardinale S, Arkin AP (2012). Contextualizing context for synthetic biology–identifying causes of failure of synthetic biological systems. Biotechnol J.

