# Supplementary material for: Resource-aware whole-cell model of division of labour in a microbial consortium for complex-substrate degradation
Source: Microb Cell Fact. 2022 Jun 14;21:115. doi: 10.1186/s12934-022-01842-0 (PMC9195437; doi:10.1186/s12934-022-01842-0)
Supplement: Supplementary file 1 — Additional file 1. Additional Tables and figures. [file 12934_2022_1842_MOESM1_ESM.docx]

**Supplementary Information**

Supplementary Table 1: Modelling framework from Weiβe et al. [33]

*Where* $\boldsymbol{x}\boldsymbol{\in}\left\{ \boldsymbol{r,et,em,q} \right\}$

| *Rates* | |
| --- | --- |
| *Transcription* | $\boldsymbol{\omega}_{\boldsymbol{x}}\left( \boldsymbol{a} \right)\boldsymbol{=}\frac{\boldsymbol{\omega}_{\boldsymbol{x}}\boldsymbol{a}}{\boldsymbol{\theta}_{\boldsymbol{x}}\boldsymbol{+a}}$  For housekeeping (q) genes only:  $\boldsymbol{\omega}_{\boldsymbol{q}}\left( \boldsymbol{a} \right)\boldsymbol{=}\frac{\boldsymbol{\omega}_{\boldsymbol{x}}\boldsymbol{a}}{\boldsymbol{\theta}_{\boldsymbol{x}}\boldsymbol{+a}}\boldsymbol{\times}\frac{\boldsymbol{1}}{\boldsymbol{1+}\left( \frac{\boldsymbol{q}}{\boldsymbol{K}_{\boldsymbol{q}}} \right)^{\boldsymbol{hq}}}$ |
| *Import* | $\boldsymbol{v}_{\boldsymbol{imp}}\boldsymbol{(et,s)=}\frac{\boldsymbol{v}_{\boldsymbol{t}}\boldsymbol{et s}}{\boldsymbol{s+}\boldsymbol{K}_{\boldsymbol{M}_{\boldsymbol{t}}}}$ |
| *Metabolism* | $\boldsymbol{v}_{\boldsymbol{cat}}\boldsymbol{(em,si)=}\frac{\boldsymbol{v}_{\boldsymbol{m}}\boldsymbol{em si}}{\boldsymbol{si+}\boldsymbol{K}_{\boldsymbol{M}_{\boldsymbol{m}}}}$ |
| *Rate of amino acid incorporation* | $\boldsymbol{\gamma}\left( \boldsymbol{a} \right)\boldsymbol{=}\frac{\boldsymbol{\gamma}_{\boldsymbol{max}}\boldsymbol{a}}{\boldsymbol{K}_{\boldsymbol{\gamma}}\boldsymbol{+a}}$ |
| *Rate of translation* | $\boldsymbol{v}_{\boldsymbol{x}}\boldsymbol{(}\boldsymbol{c}_{\boldsymbol{x}}\boldsymbol{,a)=}\frac{\boldsymbol{\gamma}\left( \boldsymbol{a} \right)}{\boldsymbol{n}_{\boldsymbol{x}}}\boldsymbol{c}_{\boldsymbol{x}}$ |
| *Intracellular molecules* | |
| *Imported glucose (si)* | $\frac{\boldsymbol{dsi}}{\boldsymbol{dt}}\boldsymbol{=}\boldsymbol{v}_{\boldsymbol{imp}}\boldsymbol{(et,s)-}\boldsymbol{v}_{\boldsymbol{cat}}\boldsymbol{(em,si)-\lambda si}$ |
| *Energy molecules (a)* | $\frac{\boldsymbol{da}}{\boldsymbol{dt}}\boldsymbol{=}\boldsymbol{n}_{\boldsymbol{s}}\boldsymbol{v}_{\boldsymbol{cat}}\boldsymbol{(em,si)-}\sum_{\boldsymbol{x}\boldsymbol{\in}\left\{ \boldsymbol{r,et,em,q} \right\}} {\boldsymbol{\gamma}\left( \boldsymbol{a} \right)\boldsymbol{c}}_{\boldsymbol{x}}\boldsymbol{-\lambda a}$ |
| *mRNA (m_x_)* | $\frac{\boldsymbol{d}\boldsymbol{m}_{\boldsymbol{x}}}{\boldsymbol{dt}}\boldsymbol{=}\boldsymbol{\omega}_{\boldsymbol{x}}\boldsymbol{(a)-}\boldsymbol{k}_{\boldsymbol{b}}\boldsymbol{m}_{\boldsymbol{x}}\boldsymbol{r+}\boldsymbol{k}_{\boldsymbol{u}}\boldsymbol{c}_{\boldsymbol{x}}\boldsymbol{+}\boldsymbol{v}_{\boldsymbol{x}}\boldsymbol{(}\boldsymbol{c}_{\boldsymbol{x}}\boldsymbol{,a)-}\boldsymbol{d}_{\boldsymbol{m}}\boldsymbol{m}_{\boldsymbol{x}}\boldsymbol{-\lambda}\boldsymbol{m}_{\boldsymbol{x}}$ |
| *Ribosome:mRNA complex (c_x_)* | $\frac{\boldsymbol{d}\boldsymbol{c}_{\boldsymbol{x}}}{\boldsymbol{dt}}\boldsymbol{=}\boldsymbol{k}_{\boldsymbol{b}}\boldsymbol{m}_{\boldsymbol{x}}\boldsymbol{r-}\boldsymbol{k}_{\boldsymbol{u}}\boldsymbol{c}_{\boldsymbol{x}}\boldsymbol{-}\boldsymbol{v}_{\boldsymbol{x}}\boldsymbol{(}\boldsymbol{c}_{\boldsymbol{x}}\boldsymbol{,a)-\lambda}\boldsymbol{c}_{\boldsymbol{x}}$ |
| *Ribosomes (r)* | $\frac{\boldsymbol{dr}}{\boldsymbol{dt}}\boldsymbol{=}\boldsymbol{v}_{\boldsymbol{r}}\left( \boldsymbol{c}_{\boldsymbol{r}}\boldsymbol{,a} \right)\boldsymbol{-\lambda r+}\sum_{\boldsymbol{x}\boldsymbol{\in}\left\{ \boldsymbol{r,et,em,q} \right\}} \boldsymbol{(}\boldsymbol{v}_{\boldsymbol{x}}\left( \boldsymbol{c}_{\boldsymbol{x}}\boldsymbol{,a} \right)\boldsymbol{-}\boldsymbol{k}_{\boldsymbol{b}}\boldsymbol{r}\boldsymbol{m}_{\boldsymbol{x}}\boldsymbol{+}\boldsymbol{k}_{\boldsymbol{u}}\boldsymbol{c}_{\boldsymbol{x}}\boldsymbol{)}$ |
| *Transport protein (et)* | $\frac{\boldsymbol{det}}{\boldsymbol{dt}}\boldsymbol{=v}_{\boldsymbol{et}}\left( \boldsymbol{c}_{\boldsymbol{et}}\boldsymbol{,a} \right)\boldsymbol{-\lambda et}$ |
| *Metabolic protein (em)* | $\frac{\boldsymbol{dem}}{\boldsymbol{dt}}\boldsymbol{=v}_{\boldsymbol{em}}\left( \boldsymbol{c}_{\boldsymbol{em}}\boldsymbol{,a} \right)\boldsymbol{-\lambda et}$ |
| *Housekeeping protein (q)* | $\frac{\boldsymbol{dq}}{\boldsymbol{dt}}\boldsymbol{=}\boldsymbol{v}_{\boldsymbol{q}}\left( \boldsymbol{c}_{\boldsymbol{q}}\boldsymbol{,a} \right)\boldsymbol{-\lambda q}$ |
| For parameter descriptions and values see Supplementary Table 2. | |

Supplementary Table 2: Parameter values

| Parameter description | Name | Value | Unit | Reference |
| --- | --- | --- | --- | --- |
| Translational threshold | K_γ_ | 7 | molecs/cell | [33] |
| Km for metabolic enzyme | Km_em_ | 1000 | molecs/cell |  |
| Km for transport enzyme | Km_et_ | 1000 | molecs/cell |  |
| Ki for housekeeping autoregulation | K_q_ | 152219 | molecs/cell |  |
| Total proteome content of cell | M | 1E+08 | amino acids |  |
| Degradation rate of mRNA | d_m_ | 0.1 | min^-1^ |  |
| Maximum translation rate | γ_max_ | 1260 | aa/min molecs |  |
| Rate of ribosome binding | k_b_ | 1 | cell/min molecs |  |
| Rate of ribosome unbinding | k_u_ | 1 | min^-1^ |  |
| Length of ribosome | n_r_ | 7549 | amino acids |  |
| Length of non-ribosomal endogenous protein | n_x_ | 300 | amino acids |  |
| Hill coefficient for housekeeping autoregulation | n_q_ | 4 | none |  |
| Nutrient efficiency | n_s_ | 0.5 | none |  |
| Transcription threshold for ribosomal genes | θ_r_ | 426.8693 | molecs/cell |  |
| Transcription threshold for non- ribosomal genes | θ_x_ | 4.379733 | molecs/cell |  |
| kcat of metabolic enzyme | ν_em_ | 5800 | min^-1^ |  |
| kcat of transport enzyme | ν_et_ | 726 | min^-1^ |  |
| Transcription rate of metabolic gene | ω_em_ | 4.139172 | molecs/min |  |
| Transcription rate of housekeeping gene | ω_q_ | 948.935 | molecs/min |  |
| Transcription rate of ribosomal gene | ω_r_ | 929.9679 | molecs/min |  |
| Transcription rate of transport gene | ω_et_ | 4.139172 | molecs/min |  |
| Length of α-amylase | n_a_ | 434 | amino acids | **Uniprot: P17654** |
| Length of glucoamylase | n_b_ | 617 | amino acids | Uniprot: Q58HN1 |
| Km for enzyme a (α-amylase) | Km_ea_ | 1140000 | molecs/cell | [47](with units converted to molecs/ cell) |
| Km for enzyme b (glucoamylase) | Km_eb_ | 68200 | molecs/cell |  |
| kcat of α-amylase reaction | ν_ea_ | 271 | min^-1^ |  |
| kcat of glucoamylase reaction | ν_eb_ | 403 | min^-1^ |  |
| Ki for glucose inhibition of α-amylase | K_i_ | 2980000 | molecs/cell |  |
| Fraction conversion of s0 | ξ | 1 | none |  |
| Molecular weight glucose | Mw_glc_ | 180 | g/mol |  |
| Molecular weight starch | Mw_s0_ | 19000 | g/mol |  |
| Ratio of cell A | N_a_ | 1 | cells | Can be adjusted based on reactor set up |
| Ratio of cell B | N_b_ | 1 | cells |  |
| Input of starch | s0_in_ | 1E+21 | molecs/min |  |
| Input of glucose | s_in_ | 1E+21 | molecs/min |  |
| Dilution rate of glucose | d_s_ | 0.001 | molecs/min |  |
| Dilution rate for starch | d_s0_ | 0.001 | molecs/min |  |
| Dilution rate for maltodextrins | d_s1_ | 0.001 | molecs/min |  |
| Dilution rate for α-amylase | d_ea_ | 0.001 | molecs/min |  |
| Dilution rate for glucoamylase | d_eb_ | 0.001 | molecs/min |  |
| Transcription rate of α-amylase | ω_ea_ | - | molecs/min | Values screened |
| Transcription rate of glucoamylase | ω_eb_ | - | molecs/min |  |

**Supplementary Table 3: Steady states of all intracellular molecules for a single cell in the monoculture and for Cell A and Cell B in the consortium**

| **Species** |  | **Molecules/cell** | | |
| --- | --- | --- | --- | --- |
|  | **Initial value** | **Monoculture single cell** | **Cell A** | **Cell B** |
| **m_ea** | 0 | 86.89994 | 71.42561 |  |
| **c_ea** | 0 | 177.796 | 252.7591 |  |
| **ea** | 0 | 16380.94 | 20130.16 |  |
| **m_eb** | 0 | 82.99183 |  | 68.70025 |
| **c_eb** | 0 | 206.6798 |  | 274.4006 |
| **eb** | 0 | 13394.28 |  | 15660.84 |
| **m_q** | 0 | 922.3015 | 661.9858 | 697.3448 |
| **c_q** | 0 | 1487.333 | 1836.807 | 1787.49 |
| **q** | 0 | 210910.1 | 222435.3 | 220860.7 |
| **m_em** | 0 | 18.85034 | 16.05385 | 16.52277 |
| **c_em** | 0 | 30.39866 | 44.54449 | 42.35249 |
| **em** | 0 | 4310.658 | 5394.288 | 5233.037 |
| **m_et** | 0 | 18.85034 | 16.05385 | 16.52277 |
| **c_et** | 0 | 30.39866 | 44.54449 | 42.35249 |
| **et** | 0 | 4310.658 | 5394.288 | 5233.037 |
| **m_r** | 0 | 88.46736 | 67.94208 | 71.05094 |
| **c_r** | 0 | 418.0531 | 573.9163 | 551.168 |
| **r** | 10 | 5.216145 | 9.408563 | 8.628292 |
| **si** | 0 | 143.0823 | 143.0823 | 143.0823 |
| **a** | 1000 | 7.840284 | 9.076709 | 8.867173 |
| **Growth rate** | | 0.015648 | 0.019581 | 0.018996 |
| **Doubling time** | | 44.29714 | 35.39852 | 36.48929 |
| **Total ribosomes** | | 2355.875 | 2761.98 | 2706.392 |
| **Total proteins** | | 249311.8 | 253363.4 | 246996.3 |
| **Heterologous/total proteins (%)** | | 11.94296 | 7.94517 | 6.34052 |

**Supplementary Table 4: Table of equations for starch-degrading monoculture and two-strain consortium**

| **Two-strain consortium** | |
| --- | --- |
| Loss of glucose from the environment | $v_{uptake}\left( et,s \right)= \frac{v_{et} s (N_{a} e{t_{cell}}_{a}+N_{b} e{t_{cell}}_{b})}{s + K_{m_{et}}}$ |
| Glucose s (if glucose is supplied externally) | $\frac{ds}{dt}=s_{in}-v_{uptake}(et,s)-d_{s} s$ |
| Glucose s (if starch is supplied externally) | $\frac{ds}{dt}=N_{b}\frac{v_{eb} eb s1}{s1+K_{m_{eb}}}-v_{uptake} \left( et,s \right)-d_{s} s$ |
| Starch s0 | $\frac{ds0}{dt}={s0_{in} - N}_{a}\frac{v_{ea} ea s0}{s0+K_{m_{ea}}} -d_{s0} s0$ |
| Intermediate s1 | $\frac{ds1}{dt}=N_{a}\frac{v_{ea} ea s0}{s0+K_{m_{ea}}} - N_{b}\frac{v_{eb} eb s1}{s1+K_{m_{eb}}}-d_{s1} s1$ |
| Imported glucose in an average Cell A | $\frac{dsi_{cell_{a}}}{dt}=\frac{v_{et}e{t_{cell}}_{a} s}{s + K_{m_{et}}}-\frac{v_{m} em_{cell_{a}} si_{cell_{a}}}{si+K_{M_{m}}}-\lambda_{cell_{a}}{si}_{cell_{a}}$ |
| Imported glucose in an average Cell B | $\frac{dsi_{cell_{b}}}{dt}=\frac{v_{et} e{t_{cell}}_{b}s}{s + K_{m_{et}}}-\frac{v_{m} em_{cell_{b}} si_{cell_{b}}}{si+K_{M_{m}}}-\lambda_{cell_{b}}si_{cell_{b}}$ |
| **Monoculture** | |
| Loss of glucose from the environment | $v_{uptake} \left( et,s \right)= \frac{v_{et} s N et}{s + K_{m_{et}}}$ |
| Glucose s (if glucose is supplied externally) | $\frac{ds}{dt}=s_{in}-v_{uptake} \left( et,s \right)-d_{s} s$ |
| Glucose s (if starch is supplied externally) | $\frac{ds}{dt}=N\frac{v_{eb} eb s1}{s1+K_{m_{eb}}}-v_{uptake} \left( et,s \right)-d_{s} s$ |
| Starch s0 | $\frac{ds0}{dt}=s0_{in} -N\frac{v_{ea} ea s0}{s0+K_{m_{ea}}} -d_{s0} s0$ |
| Intermediate s1 | $\frac{ds1}{dt}=N\frac{v_{ea} ea s0}{s0+K_{m_{ea}}} - N\frac{v_{eb} eb s1}{s1+K_{m_{eb}}}-d_{s1} s1$ |
| Imported glucose in an average monoculture cell | $\frac{dsi}{dt}=\frac{v_{et} et s}{s + K_{m_{et}}}-\frac{v_{m} em si}{si+K_{M_{m}}}-\lambda si$ |
| Species: s: glucose, s1: intermediate, s0: starch, si: imported glucose, ea: heterologous protein A (α-amylase), eb: heterologous protein B (glucoamylase), et: endogenous transport protein, em: endogenous metabolic protein (subscript denotes Cell A or Cell B which may differ in the number of intracellular molecules) | |
| All species are given as a concentration in molecules/cell assuming a constant cell volume of 1um^3^ and a constant bioreactor volume. It is assumed all species are stable with no degradation except for that provided by the desired enzyme activity. | |
| Parameters: λ: growth rate (calculated for each time point), s_in_: input of glucose by the chemostat, s0_in_: input of starch by the chemostat, N_a_: number of cells of type Cell A, N_b_: number of cells of type Cell B, N: number of cells in the monoculture (For an equivalent consortium and monoculture N = N_a_ + N_b_), v_et_: maximum rate of import by transport proteins, Km_et_: Michaelis constant for the transport reaction, v_em_: maximum rate of substrate conversion to energy by metabolic proteins, Km_em_: Michaelis constant of metabolic reaction, v_ea_: maximum rate of protein a reaction (starch to intermediate), Km_ea_: Michaelis constant of protein a reaction, v_eb_: maximum rate of protein A reaction, Km_eb_: Michaelis constant of protein B reaction, d_s_: dilution rate of glucose, d_s1_: dilution rate of intermediate, d_s0_: dilution rate of starch (assuming chemostat conditions d_s_= d_s1_= d_s0_). | |
| Refer to Supplementary Table 2 for parameter units | |

**Supplementary Figure 1: Average growth rate of cells in a consortium with different ratios of Cell A : Cell B (N_a_:N_b_)**

**
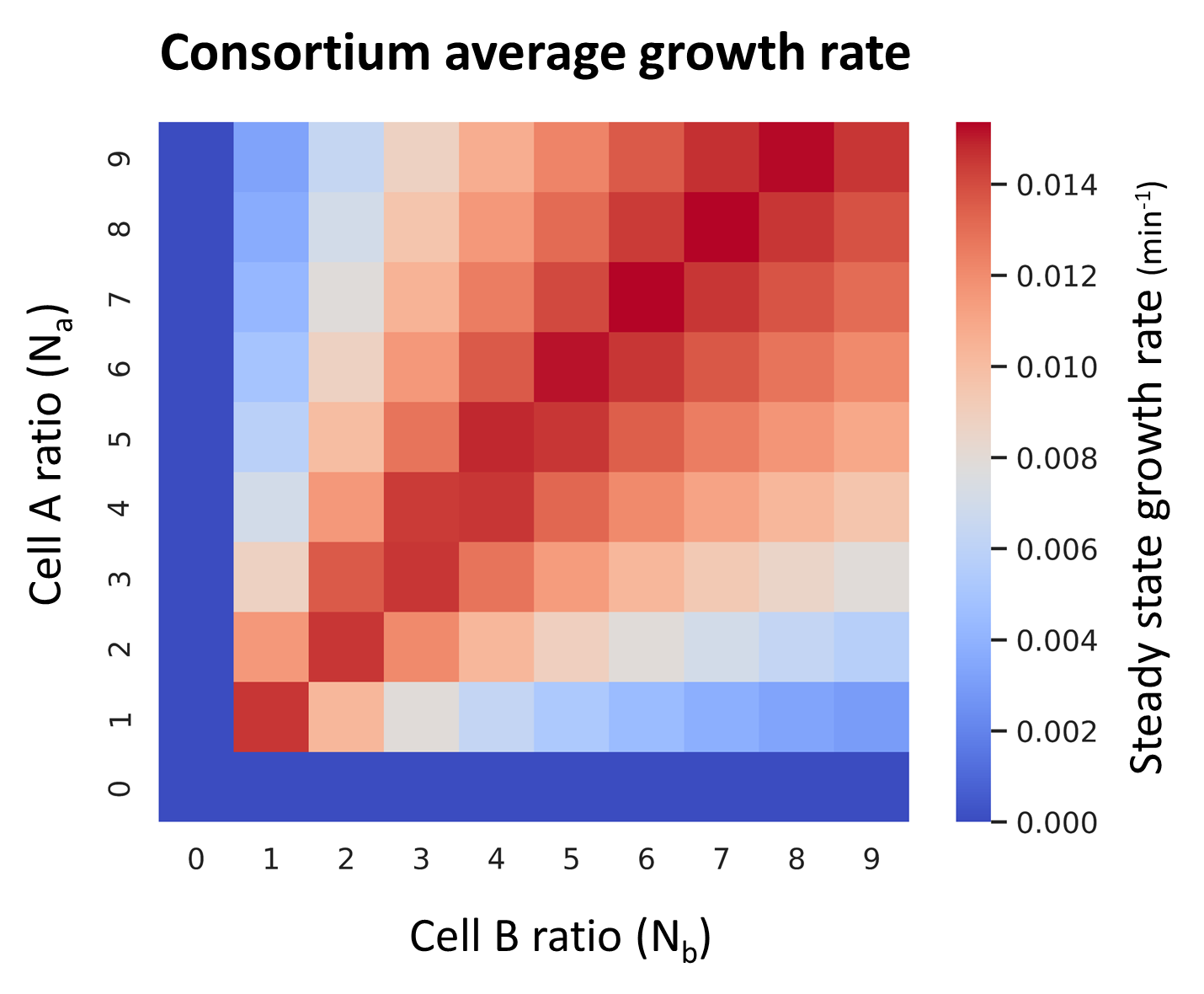
**

The ratio of the two cell types, Cell A:Cell B (N_a_:N_b_), is considered fixed and an equivalent monoculture is fixed to have N_a_ + N_b_ cells. The values of N_a_ and N_b_ in the consortium were varied and each combination was run in a simulation on starch. Here we show the steady-state growth rate of an average cell in the consortium. Growth rate is better at approximately equal ratios of the two strains, with a slight preference for Cell A at the higher transcription rates.

**Supplementary Figure 2: Difference between the steady-state production of both heterologous proteins by 2 cells of a monoculture versus 2 cells in a consortium where only one cell produces each protein**

**
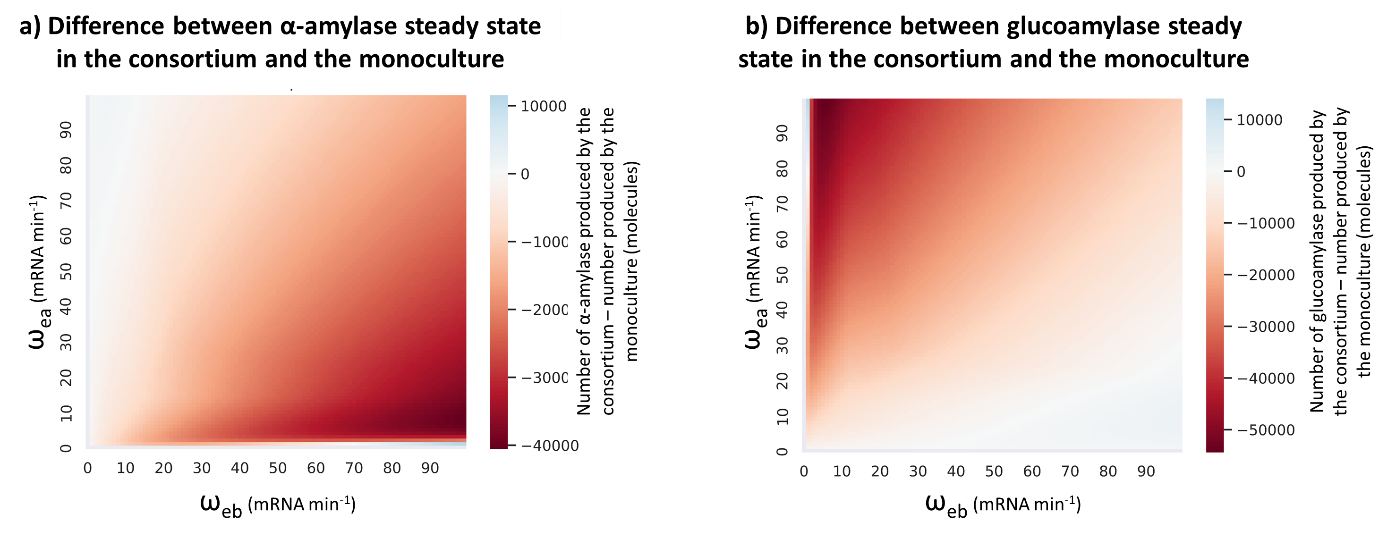
**

The whole-cell model predicts the steady state value of both heterologous amylases produced from a single average cell in a monoculture, the steady-state value of α-amylase produced by Cell A, and the steady-state value of glucoamylase produced by Cell B in the consortium. To create a fair system with equal total number of cells for the monoculture and the consortium, we assume that the consortium has 2 cells with a 1:1 ratio of the two cell types and the monoculture has 2 cells that both express both α-amylase and glucoamylase. The total amount of amylases produced by 2 cells in a monoculture will therefore be 2 times the amount predicted by the whole-cell model for one cell; allowing us to compare it to a 2-cell consortium. Here, we use our model to predict the difference between a 2-cell consortium and a 2-cell monoculture as the transcription rates of α-amylase and glucoamylase (**ω_ea_** and **ω_eb_** respectively) are varied. Difference in α-amylase is calculated as the consortium steady state minus the monoculture steady state; deeper blue means the consortium is producing more than the monoculture and deeper red means the monoculture is producing more than the consortium. The monoculture will produce more than the consortium for almost all combinations of values of the maximum transcription rates (**ω_ea_** and **ω_eb_**).

**Supplementary Figure 3: Difference between the steady state glucose level**


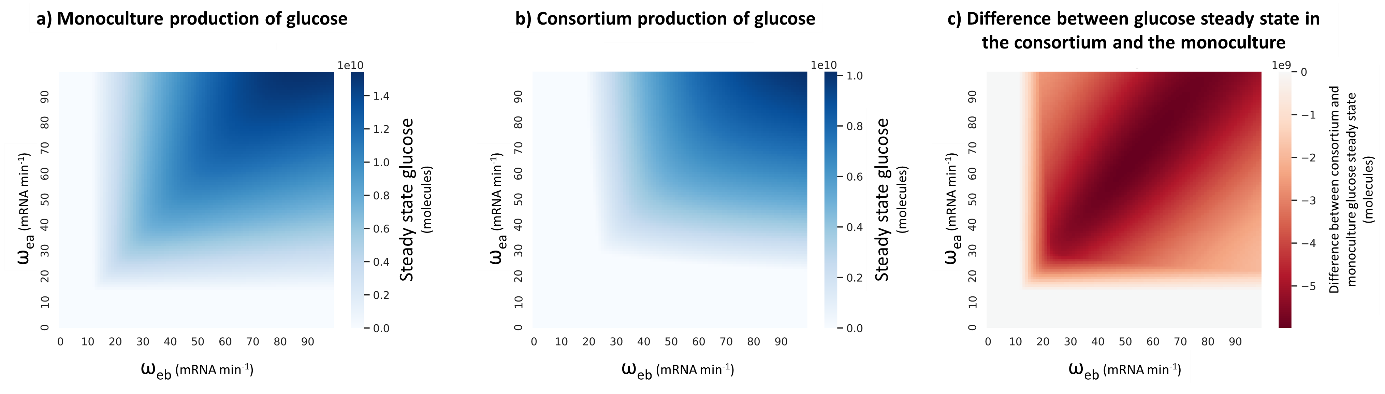


Here, we use our model to predict the difference between the steady-state level of glucose produced from starch by a 2-cell consortium (with 1 Cell A and 1 Cell B) and a 2-cell monoculture as the transcription rates of α-amylase and glucoamylase (**ω_ea_** and **ω_eb_** respectively) are varied. For the monoculture and the consortium there is a region of expression where the glucose level is 0. This is where the cell is consuming all the glucose that the amylases are creating. The monoculture reaches saturation of glucose (where there is >0 glucose at steady state, the blue region) at lower transcription rates than the consortium. Where glucose steady state is >0, the monoculture produces higher excess glucose at all combinations of the maximum transcription values (**ω_ea_** and **ω_eb_**).

**Supplementary Figure 4: The effect of different ratios of the two cell types on growth rate on starch**

**
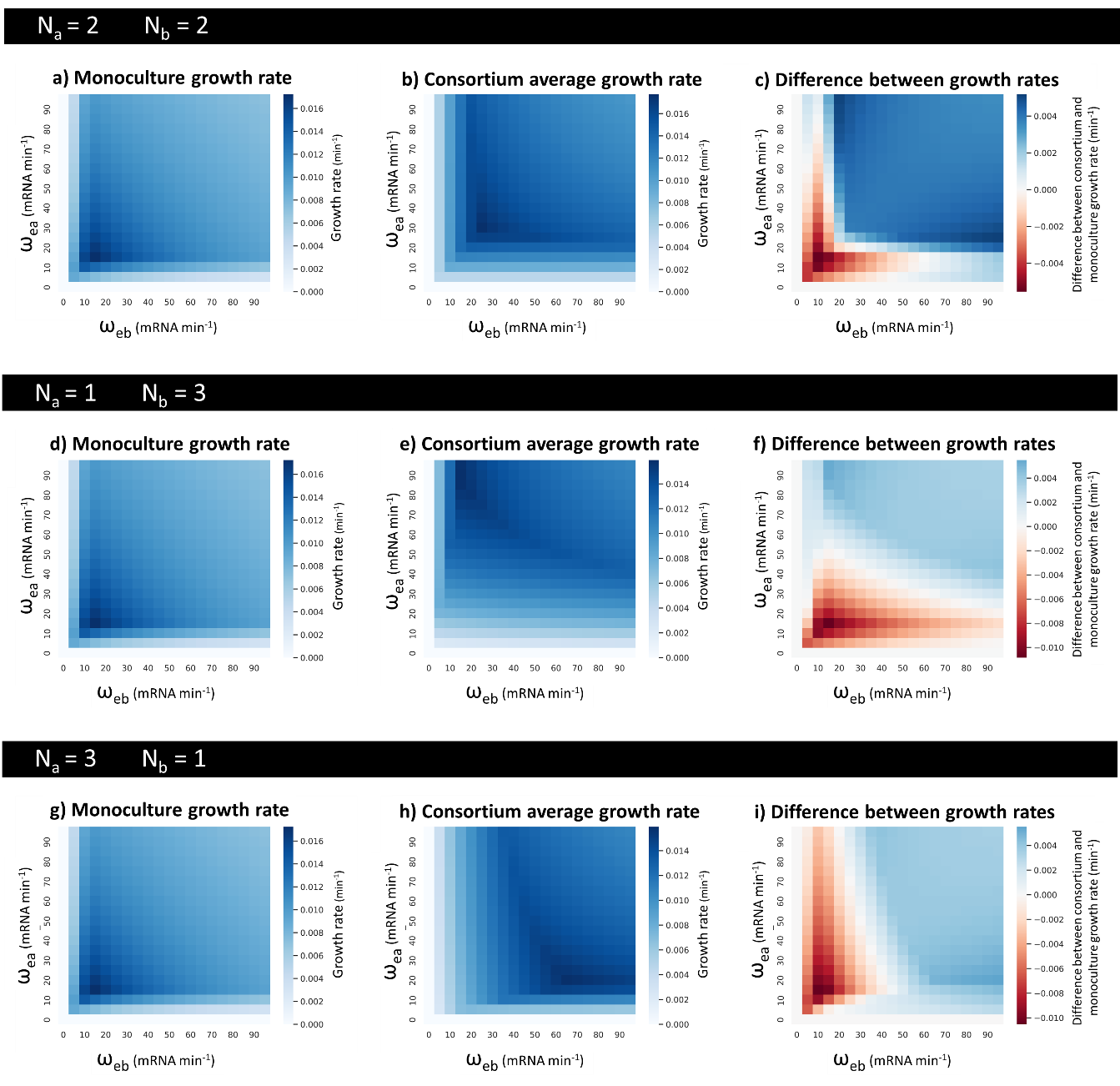
**

The ratio of the two cell types in the consortium is assumed to be stable with a ratio N_a_:N_b_. This ratio was varied to be symmetrical 2:2 (a, b, c), or asymmetrical 1:3 (d, e, f) and 3:1 (g, h, i). In all cases the equivalent monoculture was considered to have an equivalent population size of N_a_+N_b_ = 4. Maximum transcription rates of α-amylase and glucoamylase (ω_ea_ and ω_eb_ respectively) were varied. The simulation predicts the growth rates at steady state on starch as the primary carbon source. **a, d, g)** Steady-state growth rate (λ) for an average cell in a monoculture co-expressing both α-amylase and glucoamylase. **b, e, h)** Growth rate for an average cell in a two-strain consortium where Cell A expresses α-amylase and Cell B expresses glucoamylase. **c, f, i)** Comparison of monoculture and consortium growth rate (difference measured as the average growth rate of the consortium minus the monoculture growth rate).
